# Inactivation of Hepatitis A Virus and Feline Calicivirus on Model Food Contact Surfaces by Ultraviolet Light (UV-C) Systems

**DOI:** 10.3390/foods13182892

**Published:** 2024-09-12

**Authors:** Breanna Polen, Brahmaiah Pendyala, Ankit Patras, Doris H. D’Souza

**Affiliations:** 1 Department of Food Science, University of Tennessee, Knoxville, TN 37996, USA; jnn668@vols.utk.edu; 2 Department of Food and Animal Sciences, Tennessee State University, Nashville, TN 37209, USA; bpendyal@tnstate.edu (B.P.); apatras@tnstate.edu (A.P.)

**Keywords:** Hepatitis A virus, human norovirus, feline calicivirus, ultraviolet light systems, inactivation, food contact surfaces

## Abstract

Food contact surfaces can harbor and transmit pathogens leading to outbreaks. Decontamination strategies that are user- and environmentally-friendly without toxic by-product formation are needed. Novel UV-C light-emitting diode (LED) technologies are being explored to deliver the required dose to inactivate viruses in food-processing environments. The objective of this study was to compare the effects of 279 nm UV-C LED to 254 nm UV-C against hepatitis A virus (HAV) and feline calicivirus (FCV, a cultivable human norovirus surrogate) on stainless-steel, ceramic, and glass surfaces. Viruses were surface spread on sterile stainless-steel or ceramic coupons (100 μL on 2 × 2 cm^2^), or glass discs (50 μL on 1 × 1 cm^2^), air-dried, and UV-C-treated for up to 3.75 min (surface dose = 0–49.2 mJ/cm^2^ for HAV and 0–24.6 mJ/cm^2^ for FCV). Each triplicate treatment was assayed in duplicate, and data were statistically analyzed. The D_10_-values for HAV treated with UV-C at 254 nm on stainless-steel, ceramic, and glass were 9.48 ± 0.34, 14.53 ± 2.52, and 6.91 ± 1.93 mJ/cm^2^, while with UV-C LED at 279 nm were 19.53 ± 2.45, 26.05 ± 0.60, and 8.77 ± 2.08 mJ/cm^2^, respectively. The D_10_-values for FCV treated with UV-C at 254 nm on stainless-steel, ceramic, and glass were 3.65 ± 0.06, 6.25 ± 1.90, and 4.69 ± 0.03 mJ/cm^2^, while with UV-C LED at 279 nm were 7.097 ± 2.11, 8.31 ± 2.12, and 7.88 ± 0.86 mJ/cm^2^, respectively. Higher 279 nm UV-C doses were needed to inactivate HAV and FCV compared to 254 nm UV-C on the tested surfaces. Novel UV-C LED systems using appropriate doses show promise to inactivate foodborne viruses on food contact surfaces.

## 1. Introduction

Environmental sanitation is of major importance to the agricultural industry and public health sector as contamination by bacteria, viruses, parasites, or fungi can cause foodborne illnesses affecting 1 in 6 individuals in the United States annually, with 128,000 hospitalizations out of the estimated 48 million illnesses each year [1]. Though *Salmonella* is most frequently reported with 1.35 million infections and 26,500 hospitalizations in the United States annually, foodborne viruses are also increasingly reported to cause human gastroenteritis illnesses worldwide [1]. Hepatitis A virus (HAV) and human noroviruses (HuNoVs) are the epidemiologically significant foodborne viruses of human health concern [2,3]. Hepatitis A virus (HAV) is a non-enveloped, single-stranded RNA virus of the genus Hepatovirus in the family *Picornaviridae* that is transmitted through the fecal-oral route and affects the liver with symptoms that last more than a month [4]. Contact with an HAV infected individual, contamination of food by an HAV infected food handler, or contaminated surfaces can lead to illness as HAV can survive in the environment for extended periods and under low pH conditions [5]. Although, effective HAV vaccinations were introduced in 1995 with a decrease in number of HAV outbreaks, they continue to occur worldwide [6]. As of August 2023, in the U.S. alone, HAV outbreaks were reported in 37 states totaling 44,903 cases, 27,435 hospitalizations, and 423 deaths linked to person-to-person contact [2]. 

While HAV surveillance is important due to the severity and length of disease symptoms, HuNoV illnesses are the most frequently reported foodborne illness with 21 million illnesses occurring annually in the U.S. [7]. HuNoV’s belong to the *Caliciviridae* family and like HAV are also non-enveloped with a positive-sense, single-stranded RNA enclosed in a capsid, but have a “cup-like” shape and though are transmitted in a similar manner as HAV, have a short incubation period [8]. They are also environmentally stable and possess an infection dose between 18 and 2,800 particles which makes prevention of its spread extremely difficult, especially in the absence of commercially available vaccines [9]. Although HuNoV infection is typically self-limiting in healthy individuals, severe health complications can occur in immunocompromised individuals, the elderly, and young children [8]. Foodborne HuNoV outbreaks continue to occur and a multistate HuNoV outbreak linked to raw oysters was reported between November and December of 2022 in Texas, U.S. [7] and in British Columbia with 192 illnesses as of June 2022 [7]. Since all genogroups of HuNoV’s cannot reproducibly be cultivated in the laboratory at high titers for inactivation studies, cultivable surrogates including feline calicivirus (FCV-F9), murine norovirus (MNV-1) and Tulane virus (TV) have been used [10,11,12].

The importance of sanitation of food contact surfaces in the prevention of foodborne outbreak spread is well-recognized within the food industry. Commonly used chemical disinfectants include chlorine, quaternary ammonium, hydrogen peroxide, and peracetic acid [13,14,15], but can generate hazardous waste and harmful by-products. An alternate surface disinfection approach is the application of ultraviolet (UV) light-C technology in the range of 200 to 280 nm, traditionally using low-pressure mercury lamps (typically at 254 nm), for inactivation of microorganisms [16,17]. UV-C damages DNA and RNA due to photochemical changes, cross-linking, and oxidative damage and prevents DNA replication [18]. The genetic material within viral capsids are strong absorbers of UV radiation, especially near the 254 nm wavelength, whereas other viral components such as proteins are minor absorbers of UV radiation at this wavelength [19]. Research indicates that UV-C at 254 nm inactivation follows a dose-dependent relationship with varying effects depending on the type of microorganism [20]. Previous studies showed that dried FCV on stainless-steel discs was completely inactivated (>5.0 log) at a 254 nm UV-C dose of 60 mJ/cm^2^ [21]. Another study reported a 2.6 log reduction of HAV on stainless-steel surfaces with 300 mJ/cm^2^ using 254 nm UV-C [22].

However, more recently, UV-C light emitting diodes (LEDs) have become widely studied as a decontamination approach with energy efficient inactivation of microorganisms [23,24,25]. LEDs possess intrinsic properties regarding optical features, are more sustainable, portable, inexpensive with low heat emission, have increased wavelength diversity, do not require a warmup time, and without hazardous waste generation due to their lack of mercury as opposed to traditional UV-C mercury containing lamps [26]. UV-C LEDs can be designed to have a peak wavelength close to absorption maxima of DNA and RNA or proteins [23].

There is limited research on the use of UV-C LED at varying wavelengths such as 279 nm for the inactivation of foodborne viruses on contact surfaces. Previously, the HuNoV surrogate, feline calicivirus (FCV-F9) on stainless-steel surfaces was shown to be reduced by 3 log PFU/disc using UV-C LED technology at 269 nm at a dose of 22.5 mJ/cm^2^ [27]. Furthermore, the dose required to achieve a 1 log or 90% reduction (D_10_-value) of FCV on Formica coupons was reported to be 23.37 ± 0.91 mJ/cm^2^ using UV-C LED at 279 nm and 9.97 ± 2.44 mJ/cm^2^ using UV-C at 254 nm [28]. This same study also showed that HAV on Formica coupons had similar D_10_-values using both traditional UV-C at 254 nm (D_10_ = 12.40 ± 1.15 mJ/cm^2^) and UV-C LED at 279 nm (D_10_ = 12.39 ± 0.70 mJ/cm^2^) [28]. Taken together, UV-C LED at 279 nm shows promise for inactivation of viruses on surfaces with its advantages over traditional UV-C at 254 nm. Therefore, this aimed to evaluate the effectiveness of UV-C LED at 279 nm in comparison to traditional UV-C at 254 nm for the inactivation of HAV and the cultivable human norovirus surrogate, FCV-F9 dried on stainless-steel, ceramic and glass surfaces as model food contact surfaces.

## 2. Materials and Methods

### 2.1. Animal Host Cell Lines for HAV and FCV Propagation

Fetal rhesus monkey kidney cells (FRHK-4) were used as host cells for the propagation of HAV-HM175 generously provided by Dr. Kalmia Kniel’s laboratory (University of Delaware), and Crandell-Reese Feline Kidney (CRFK) cells were used as host cells for the propagation of FCV-F9, obtained from the American Type Culture Collection (ATCC, Manassas, VA, USA), as reported in our earlier studies [11,29]. Dulbecco’s Modified Eagle Medium (DMEM-F12) containing 10% fetal bovine serum (FBS) and 1% Penicillin Streptomycin (Pen-Strep) (PS; Fisher Scientific, Pittsburgh, PA, USA) were used to maintain and propagate both cell lines.

### 2.2. HAV and FCV Propagation

Previously published protocols were used to propagate HAV and FCV [11,29,30]. Briefly, 2 mL of HAV (strain HM175), generously provided by Dr. Kalmia Kniel’s laboratory (University of Delaware), was added to confluent FRHK-4 cells within sterile 175 cm^2^ cell-culture flasks along with 8 mL DMEM-F12 containing 2% FBS and 1% Pen-Strep and incubated at 37 °C for three hours in a CO_2_ incubator with 5% CO_2_. This was followed by the addition of 10 mL DMEM-F12 containing 10% FBS and 1% Pen-Strep. The infected flasks were then incubated for five to seven days, within a water-jacketed incubator under 5% CO_2_. Once cytopathic effects were observed, the infected flasks were frozen within a −80 °C freezer and freeze-thawed thrice, centrifuged at 5000 rpm for 10 min and filtered through a 0.2-micron filter to be stored back at −80 °C for subsequent use as additional HAV stock as reported before [12].

Similarly, to the cultivation of the previously mentioned FRHK-4 cells and infection of HAV, confluent CRFK cells within sterile 175 cm^2^ cell-culture flasks were infected with 2 mL of FCV-F9 stock (purchased from ATCC) and 8 mL DMEM with 2% FBS and 1% Pen-Strep and incubated at 37 °C for three hours, followed by the addition of 10 mL DMEM-F12 containing 10% FBS and 1% Pen-Strep and incubated for three to five days, within a water-jacketed incubator under 5% CO_2_ until cytopathic effects were observed. The infected flasks were then freeze-thawed thrice, centrifuged and filtered through a 0.2-micron filter to be stored back at −80 °C for subsequent use as additional FCV-F9 stock as reported earlier [11]. 

### 2.3. UV-C LED (279 nm) Treatment of HAV and FCV Inoculated and Dried on Stainless-Steel Coupons, Ceramic Coupons, and Glass Discs

Stainless-steel coupons (Biosurface Technologies, via Fisher Scientific, Pittsburgh, PA, USA, type 304, finish 2B; 2 × 2 cm^2^) or ceramic tiles/coupons (Home Depot; 2 × 2 cm^2^) or glass discs (Biosurface Technologies; 1 × 1 cm^2^) were surface rinsed with ethanol, dried, wrapped with aluminum foil and sterilized by autoclaving. These sterile coupons or discs were placed within sterile petri dishes within a biosafety hood (254 nm UV-C mercury lamp, Labconco Purifier Class II Biosafety cabinet, 36208 020421542 A, Kansas City, MO, USA) and surface decontaminated using 254 nm UV-C light for 10 min as described in earlier studies [28]. Then, 100 μL of either HAV (~5.5 log PFU/mL) or FCV (~6.0 log PFU/mL) in cell-culture grade phosphate buffered saline (pH 7.2, Fisher Scientific) were aseptically spread onto individual sterile coupons (2 × 2 cm^2^ area of stainless- steel or ceramic) or 50 μL were spread on glass discs (1 × 1 cm^2^ area) and allowed to dry within the biosafety hood for another 10 min for stainless-steel and ceramic coupons or overnight for glass discs (at an ambient temperature of 23 °C and 43% relative humidity) and exposed to various UV doses/treatment times as described below.

UV-C LED (MD 1016-1, Irtronix, Torrence, CA, USA) emits at a peak wavelength of 279 nm. For treatments, the sum of the 10–12 nm full width at half maximum output irradiance and corresponding wavelength absorption spectra were considered for average delivered UV dose irradiance calculation. The exposures were conducted for up to 2.5 min for HAV on stainless-steel coupons using 30 s intervals (0–150 s), 3.75 min for HAV on ceramic coupons using 45 s intervals (0–225 s), and up to 1.0 min for HAV on glass using 10 s intervals until 40 s, followed by a 20 s interval (0–60 s). Treatments up to 1.25 min for FCV on stainless-steel coupons using 15 s intervals (0–75 s), 2.5 min for FCV on ceramic coupons using 30 s intervals (0–150 s), and up to 1.0 min for FCV on glass using 10 s intervals until 40 s, followed by a 20 s interval (0–60 s) (279 nm, 6.5 cm from sample, Surface irradiance = 0.328 mW/cm^2^, HAV surface dose = 0–49.2 mJ/cm^2^ on stainless-steel, 0–73.8 mJ/cm^2^ on ceramic, 0–19.68 mJ/cm^2^ on glass; FCV surface dose = 0–24.6 mJ/cm^2^ for stainless-steel, 0–49.2 mJ/cm^2^ on ceramic, and 0–19.68 mJ/cm^2^ on glass). Each experiment was replicated three times for each treatment condition/exposure time/UV dose. 

Viruses from the inoculated control (0–min) ceramic and stainless-steel coupons as well as the UV-C LED exposed treated coupons were then aseptically recovered using 750 μL DMEM-F12 containing 2% FBS and 1% Pen-strep that served as an eluant for HAV or FCV and aseptically added to sterile 2 mL centrifuge tubes. Viruses from the inoculated control (0–min) glass discs as well as the UV-C LED exposed treatments were aseptically recovered by placing the glass disc within a 15 mL sterile centrifuge tube containing 9 mL DMEM-F12 with 2% FBS and 1% Pen-strep that served as an eluant for HAV and FCV. The recovered viruses were ten-fold serially diluted using DMEM-F12 containing 2% FBS and 1% Pen-Strep. The viral infectivity was determined using viral plaque assays in duplicate for each ten-fold dilution for each treatment type and treatment time/dose. 

### 2.4. UV-C (254 nm) Treatment of HAV and FCV Inoculated and Dried on Stainless-Steel Coupons, Ceramic Coupons and Glass Discs

Similar to UV-C LED treatments of viruses, sterile stainless-steel and ceramic coupons or glass discs were placed within sterile petri dishes within a biosafety hood (254 nm, Labconco Purifier Class II Biosafety cabinet, 36208 020421542 A, Kansas City, MO, USA) for surface decontamination using 254 nm UV-C light for 10 min and 100 µL of either HAV (~5.5 log PFU/mL) or FCV (~6.0 log PFU/mL) were spread on stainless-steel or ceramic discs or 50 μL of each virus were aseptically spread on glass discs and allowed to dry within the biosafety hood for 10 min for the coupons and overnight for the discs (at an ambient temperature of 23 °C and 43% relative humidity). The UV-C at 254 nm lamp was turned on for a 10-min warm-up time before treatments were conducted. UV-C hood light treatments at 254 nm (254 nm, 55.88 cm/22 inches from sample, Surface irradiance = 0.217 mW/cm^2^, HAV surface dose = 0–32.55 mJ/cm^2^ for stainless-steel, 0–48.83 mJ/cm^2^ for ceramic, 0–13.02 mJ/cm^2^ for glass; FCV surface dose = 0–16.28 mJ/cm^2^ for stainless-steel and ceramic, and 0–13.02 mJ/cm^2^ for glass) were conducted for up to 2.5 min for HAV on stainless-steel coupons using 30 s intervals (0–150 s), 3.75 min for HAV on ceramic coupons using 45 s intervals (0–225 s), treatments for up to 1.25 min for FCV on stainless-steel coupons and ceramic coupons using 15 s intervals (0–75 s), and 1 min treatment for both HAV and FCV on glass discs with 10 s intervals until 40 s followed by a 20 s interval until 1 min (0–60 s). Using the same methods reported above for UV-C LED in Section 2.3, viruses from both the 0–min control and the UV-C 254 nm treatments were recovered, and viral plaque assay was performed in duplicate for each of the three replicates for each treatment condition.

### 2.5. Infectious Plaque Assays

Standard plaque assays in 6-well plates containing confluent host cells were used to determine the infectivity of both viruses [28]. Briefly, the confluent host FRhK-4 cells were infected with 500 µL of ten-fold serially diluted recovered HAV from control and treated coupons. After infection, the plates were incubated at 37 °C and 5% CO_2_ for 2.5 h. Then, the media was aspirated, and the infected cells were overlaid with 2 mL per well of a 1:1 ratio of 1.5% Noble agar and HAV overlay medium [28]. After the overlay, the plates were stored for 72–120 h at 37 °C and 5% CO_2_ until visualization and enumeration of plaques, and recovered viruses were reported as log PFU/mL [12,28].

Similarly, confluent host CRFK cells in 6-well plates were infected with 500 µL of ten-fold serially diluted FCV recovered from control and treated coupons. After infection, the plates were incubated at 37 °C and 5% CO_2_ for 2.5 h, and the media was then aspirated. The infected cells were overlaid with 2 mL per well of a 1:1 ratio of 1.5% Noble agar and 2× FCV overlay medium as reported earlier [11,28]. After the overlay, the plates were stored for 72–120 h in the CO_2_ incubator at 37 °C and 5% CO_2_ until visualization and enumeration of plaques.

### 2.6. UV-C Dose Calculation 

Surface irradiances for the UV-C LED at 279 nm device were measured and calculated using a highly sensitive spectrophotometer (QE Pro series, Ocean Optics, Dunedin, FL, USA) within the exposure area, as discussed previously [31,32]. Surface irradiances for the UV-C at 254 nm system were measured using an International Light Technologies (ILT) research radiometer (ILT5000, SED033/F/W, International Light Technologies, Peabody, MA, USA) which was equipped with a light meter, sensor, filter, optic, and calibration. The surface dose was calculated using the UV intensity and various exposure time (s) as reported in previous studies [24,28,32]. The calculated UV intensity for the UV-C (254 nm) was 0.217 mW/cm^2^ and the UV intensity of the UV-C LED was 0.328 mW/cm^2^, as calculated using Equation (1) [31].

Surface UV-C dosage (mJ/cm^2^) = UV intensity (mW/cm^2^) × exposure time (s) (1)

### 2.7. Surface Roughness Measurements

For each surface type, the surface roughness was measured and reported using a portable tester SJ-210 by Mitutoyo (Sakado, Takatsu-Ku, Kawasaki, Kanagawa, Japan) at Tennessee State University (Nashville, TN, USA) as described in previous studies, using the SJ-210 V.1.210 software with standard configurations [32]. Briefly, as described earlier, the constant measurement conditions (Lc-0.1 in, Ls-0.000813 cm, Sampling lengths (N)-4, Prelength-OFF, Pitch-0.000150 cm) were used for all the measurements and with a 0.0508 cm/s probe (sensor) speed. Similar to previous reports, the surface roughness (μm) of stainless-steel and ceramic coupons as well as glass discs were measured for an average of 3 sampling lengths/periods [32].

### 2.8. Statistical Analysis

A two-way ANOVA statistical test with Tukey’s adjustments (*p* < 0.05) using JMP v.17 was used to analyze the significant differences between the systems, surfaces, and their interactions for each tested virus. No random effects were introduced. A Shapiro-Wilk W and QQ normality plots were used to evaluate the normality of ANOVA residuals. All statistical assumptions regarding normality were met. Excel® was used to determine the D_10_-values from linear models as reported earlier [28].

## 3. Results

### 3.1. Inactivation of HAV and FCV on Stainless-Steel Coupons Using UV-C at 254 nm

**Figure 1:**
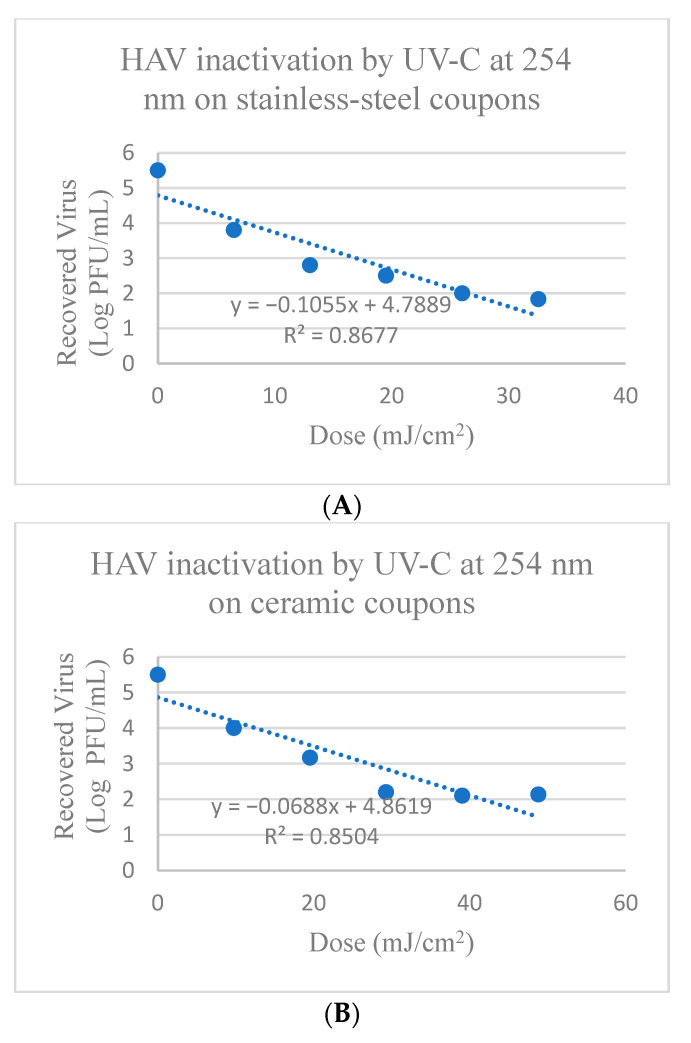

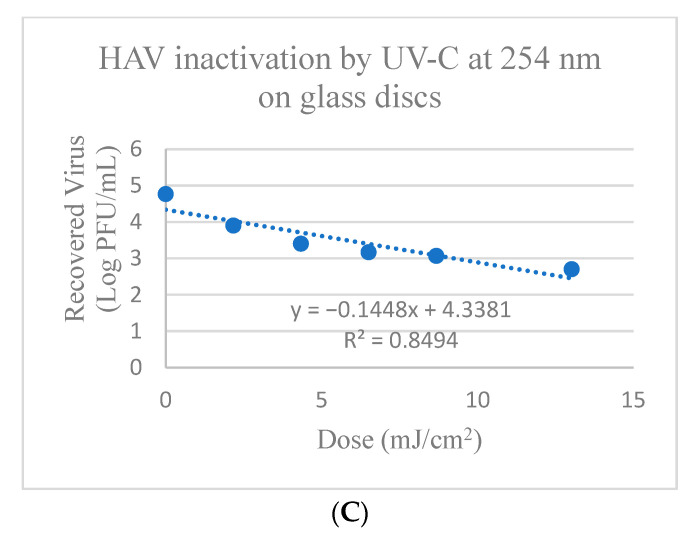
Inactivation of HAV by UV-C at 254 nm on (**A**) Stainless-steel; (**B**) Ceramic; and (**C**) Glass surfaces. Corresponding Linear 1D_10_-values for HAV on stainless-steel surfaces = 9.48 mJ/cm^2^; Linear 2D_10_ = 18.96 mJ/cm^2^; Linear 3D_10_ = 28.44 mJ/cm^2^; Linear 1D_10_-values for HAV on ceramic surfaces = 14.53 mJ/cm^2^; Linear 2D = 29.07 mJ/cm^2^; Linear 3D = 43.60 mJ/cm^2^; and on glass discs linear 1D_10_-values = 6.91 mJ/cm^2^; Linear 2D_10_ = 13.81 mJ/cm^2^; Linear 3D_10_ = 20.72 mJ/cm^2^.

HAV on stainless-steel coupons treated with UV-C at 254 nm showed reduction between 1.26 to 3.63 log PFU after treatment times of 0.5 min to 2.5 min (corresponding to doses of 6.51–32.55 mJ/cm^2^), respectively (Table S1 and Figure 1). HAV titers were significantly reduced (*p* < 0.05) by 1.26 ± 0.07 log PFU compared to the control after treatment for 0.5 min and was further reduced by 2.53 ± 0.05 log PFU after 1 min treatment and by 3.50 ± 0.16 log PFU after 2 min treatment, followed by a tailing effect in reduction that occurred past the 2 min treatment with UV-C at 254 nm (Figure 1). The calculated D_10_-value (mJ/cm^2^) for the dose required to achieve a 1 log reduction of HAV using 254 nm UV-C on stainless-steel surfaces was 9.48 ± 0.34 mJ/cm^2^ as shown in Table 1. 

**Figure 2:**
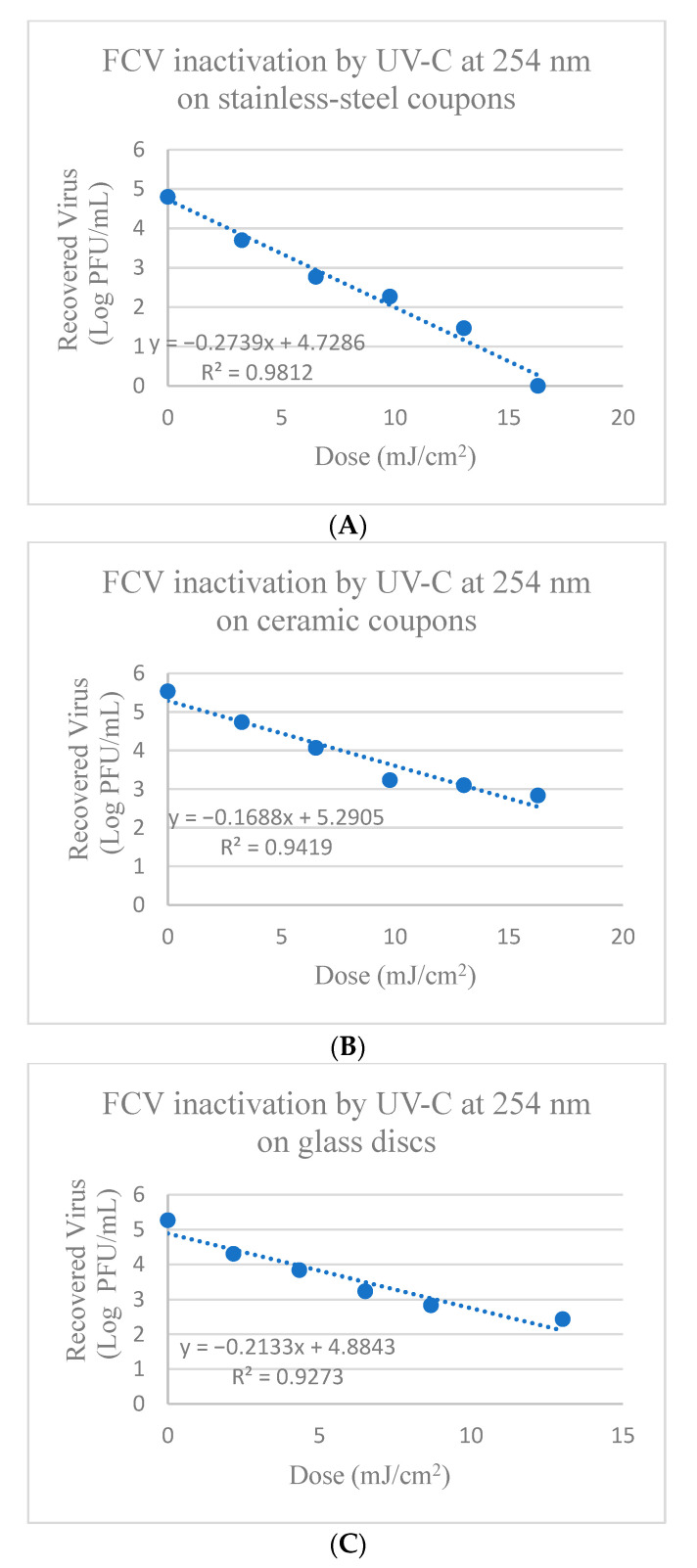
Inactivation of FCV by UV-C at 254 nm on (**A**) Stainless-steel; (**B**) Ceramic; and (**C**) Glass surfaces. Corresponding Linear 1D_10_-values for FCV on stainless-steel = 3.65 mJ/cm^2^; Linear 2D_10_ = 7.3 mJ/cm^2^; Linear 3D_10_ = 10.95 mJ/cm^2^; Linear 1D-values for FCV on ceramic = 5.92 mJ/cm^2^; Linear 2D = 11.85 mJ/cm^2^; Linear 3D = 17.78 mJ/cm^2^; and Linear 1D_10_-values for FCV on glass surfaces = 4.69 mJ/cm^2^; Linear 2D = 9.38 mJ/cm^2^; Linear 3D = 14.06 mJ/cm^2^.

FCV on stainless-steel coupons treated with UV-C at 254 nm showed reduction between 1.1 to 4.8 log PFU after 0.25 to 1.25 min (corresponding to doses of 3.26–16.28 mJ/cm^2^), respectively (Table S2 and Figure 2). There was significant reduction in FCV titers compared to the control after each treatment time (*p* ≤ 0.05). The calculated D_10_-value (mJ/cm^2^) for 1 log reduction of FCV on stainless-steel coupons was 3.65 ± 0.06 mJ/cm^2^ as shown in Table 2.

### 3.2. Inactivation of HAV and FCV on Ceramic Coupons Using UV-C at 254 nm

HAV on ceramic coupons treated with UV-C at 254 nm after 0.75 min to 3.75 min (corresponding to doses of 9.77–48.83 mJ/cm^2^) showed reduction between 1.52 to 3.35 log PFU, respectively (Table S3 and Figure 1). There was significant reduction in HAV titers after 0.75 min treatment of 1.52 ± 0.12 log PFU, and after 1.5 min and 2.25 min treatment of 2.32 ± 0.11 and 3.30 ± 0.13 log PFU, respectively (*p* ≤ 0.05), though tailing effects in reduction were observed after 2.25 min of exposure (Figure 1). The calculated D_10_-value (mJ/cm^2^) for 1 log reduction of HAV on ceramic coupons was 14.53 ± 2.52 mJ/cm^2^ as shown in Table 1.

FCV on ceramic coupons treated with UV-C at 254 nm showed reduction between 0.77 to 2.67 log PFU after treatment for 0.25 min to 1.25 min (corresponding to doses of 3.26–16.28 mJ/cm^2^), respectively (Table S4 and Figure 2). There was significant reduction in FCV titers of 0.77 ± 0.07 log PFU after 0.25 min treatment, 1.46 ± 0.03 log PFU reduction after 0.5 min treatment, and 2.28 ± 0.07 log PFU reduction after 0.75 min treatment (*p* ≤ 0.05), though the reduction tailed after 0.75 min of exposure (Figure 2). The calculated D_10_-value (mJ/cm^2^) for 1 log reduction of FCV on ceramic coupons was 6.25 ± 1.90 mJ/cm^2^ using UV-C at 254 nm, as shown in Table 2. 

### 3.3. Inactivation of HAV and FCV on Glass Discs Using UV-C at 254 nm

HAV on glass discs treated with UV-C at 254 nm for 0.17 to 1.0 min (corresponding to doses of 2.17–13.02 mJ/cm^2^) showed reduction between 0.85 to 2.09 log PFU, respectively (Table S5 and Figure 1). There was significant reduction in HAV titers of 0.85 ± 0.02 log PFU after 0.17 min treatment, with 1.3 ± 0.10 and 2.09 ± 0.10 log PFU reduction after 0.33- and 1-min treatment, respectively (*p* ≤ 0.05). The calculated D_10_-value (mJ/cm^2^) to achieve a 1 log reduction of HAV on glass discs was 6.91 ± 1.93 mJ/cm^2^ using UV-C at 254 nm, as shown in Table 1. 

FCV on glass discs showed reduction between 0.98 to 2.86 log PFU after treatment with UV-C at 254 nm for 0.17 to 1.0 min (corresponding to doses of 2.17–13.02 mJ/cm^2^), respectively (Table S6 and Figure 2). There was significant reduction in FCV titers after all times of exposure (0.17, 0.33, 0.5, 0.67 min) until 0.67 min, where tailing reduction began (*p* ≤ 0.05) (Figure 2). The calculated D_10_-value (mJ/cm^2^) to achieve a 1 log reduction of FCV on glass discs was 4.69 ± 0.03 mJ/cm^2^ using UV-C at 254 nm, as shown in Table 2.

### 3.4. Inactivation of HAV and FCV on Stainless-Steel Coupons Using UV-C at 279 nm

**Figure 3:**
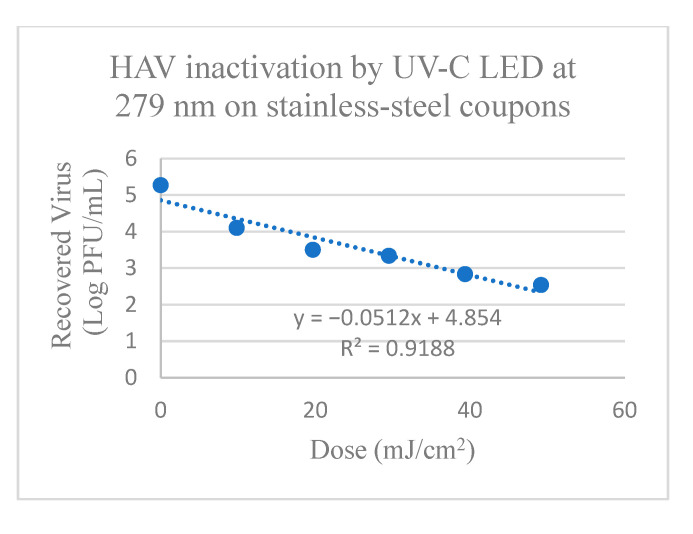

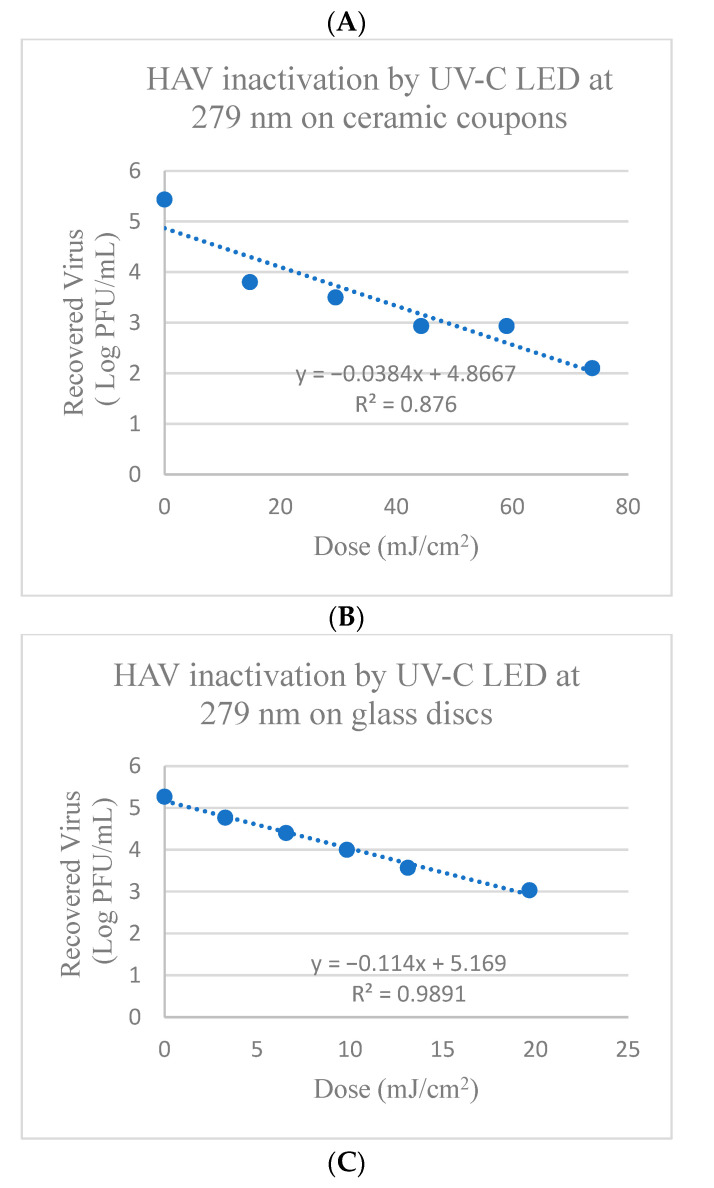
Inactivation of HAV by UV-C LED at 279 nm on (**A**) Stainless-steel; (**B**) Ceramic; and (**C**) Glass surfaces. Corresponding Linear 1D_10_-values for HAV on stainless-steel surfaces19.53 mJ/cm^2^; Linear 2D_10_ = 39.06 mJ/cm^2^; Linear 3D_10_ = 58.59 mJ/cm^2^; Linear 1D_10_-values for HAV on ceramic surfaces = 26.04 mJ/cm^2^; Linear 2D_10_ = 52.08 mJ/cm^2^; Linear 3D_10_ = 78.13 mJ/cm^2^; and on glass discs linear 1D_10_-values = 8.77 mJ/cm^2^; Linear 2D_10_ = 17.54 mJ/cm^2^; Linear 3D_10_ = 26.31 mJ/cm^2^.

HAV on stainless-steel coupons showed reduction between 1.2 to 2.75 log PFU after treatment for 0.5 min to 2.5 min (corresponding to doses of 9.84–49.2 mJ/cm^2^) with UV-C LED at 279 nm (Table S1 and Figure 3). There was significant reduction in HAV titers of 1.20 ± 0.07 log PFU after 0.5 min treatment from the control, with 1.96 ± 0.02 log PFU reduction after 1.5 min treatment and further reduction of 2.75 ± 0.15 log PFU/mL after 2.5 min treatment. The calculated D_10_-value (mJ/cm^2^) required to achieve a 1 log reduction of HAV on stainless-steel coupons was 19.53 ± 2.45 mJ/cm^2^ using UV-C at 279 nm, as shown in Table 1. 

**Figure 4:**
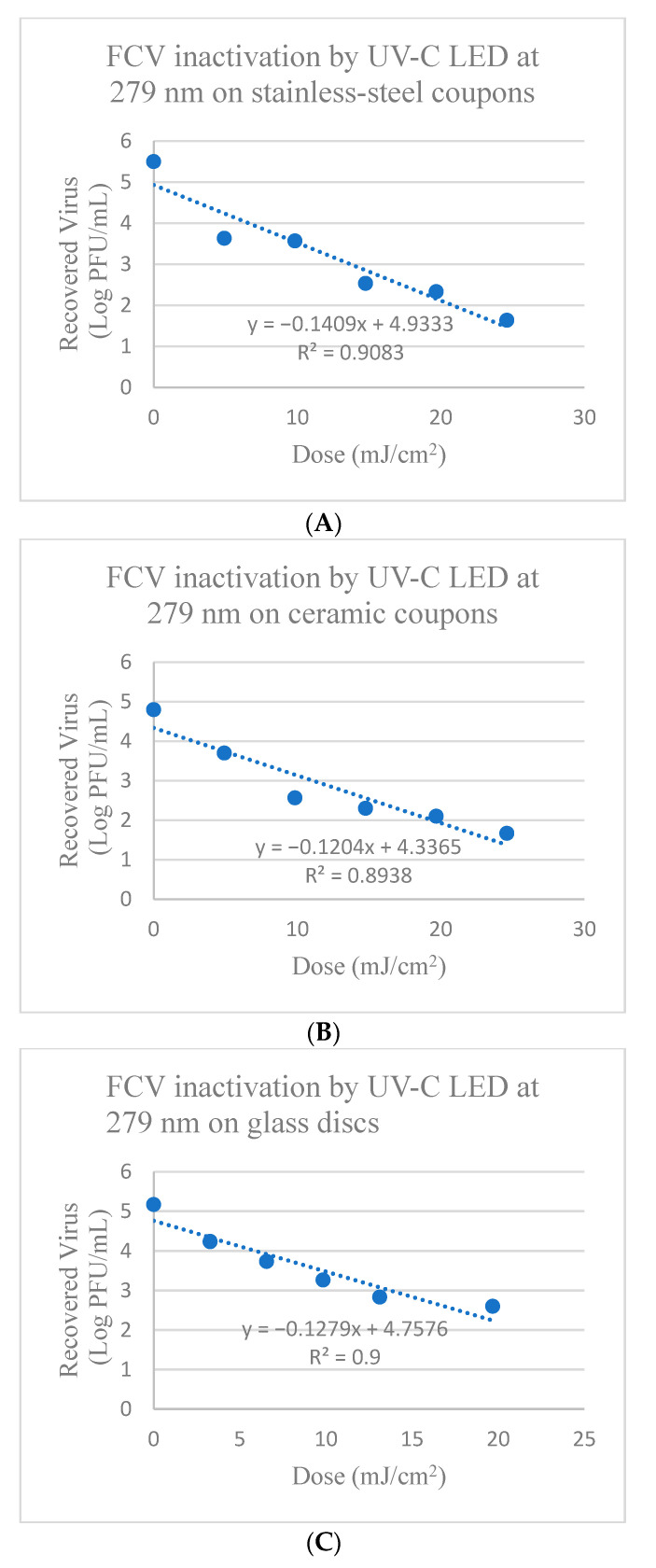
Inactivation of FCV by UV-C LED at 279 nm on (**A**) Stainless-steel; (**B**) Ceramic; and (**C**) Glass surfaces. Corresponding Linear 1D_10_-values for FCV on stainless-steel surfaces = 7.1 mJ/cm^2^; Linear 2D_10_ = 14.2 mJ/cm^2^; Linear 3D_10_ = 21.3 mJ/cm^2^; Linear 1D_10_-values for FCV on ceramic surfaces = 8.31 mJ/cm^2^; Linear 2D_10_ = 16.61 mJ/cm^2^; Linear 3D_10_ = 24.92 mJ/cm^2^; and on glass discs linear 1D_10_-values = 7.82 mJ/cm^2^; Linear 2D_10_ = 15.64 mJ/cm^2^; Linear 3D_10_ = 23.46 mJ/cm^2^.

FCV on stainless-steel coupons showed reduction between 1.93 to 3.89 log PFU/mL after treatment with UV-C LED at 279 nm for 0.25 to 1.25 min (corresponding to doses of 4.92–24.6 mJ/cm^2^) (Table S2 and Figure 4). There was significant reduction in FCV titers after 0.25 min of 1.93 ± 0.05 log PFU, with 2.99 ± 0.11 log PFU reduction after 0.75 min, followed by further reduction of 3.89 ± 0.19 log PFU after 1.25 min (*p* ≤ 0.05). The calculated D_10_-value (mJ/cm^2^) to achieve a 1 log reduction of FCV on stainless-steel coupons was 7.097 ± 2.11 mJ/cm^2^ using UV-C LED at 279 nm, as shown in Table 2.

### 3.5. Inactivation of HAV and FCV on Ceramic Coupons Using UV-C at 279 nm

HAV on ceramic coupons showed reduction between 1.63 to 3.37 log PFU after treatment with UV-C LED at 279 nm for 0.75 min to 3.75 min (corresponding to doses of 14.76–73.8 mJ/cm^2^), respectively (Table S3 and Figure 4). There was significant reduction after 0.75 min of 1.63 ± 0.17 log PFU from the control with further significant reduction of 2.52 ± 0.11 log PFU after 2.25 min and with 3.37 ± 0.10 log PFU reduction again after 3.75 min treatment. The calculated D_10_-value (mJ/cm^2^) for 1-log inactivation of HAV on ceramic coupons using UV-C at 279 nm was 26.05 ± 0.60 mJ/cm^2^ as shown in Table 1.

FCV on ceramic coupons showed reduction between 1.1 to 3.12 log PFU after treatment treated with UV-C LED at 279 nm for 0.5–2.5 min (corresponding to doses of 9.84–49.2 mJ/cm^2^) (Table S4 and Figure 4). There was significant reduction in FCV titers after treatment for 0.5 min of 1.10 ± 0.04 log PFU, with 2.51 ± 0.07 log PFU after 1.5 min treatment and 3.12 ± 0.11 log PFU after 2.5 min treatment (*p* ≤ 0.05). The calculated D_10_-value (mJ/cm^2^) for FCV on ceramic coupons was 8.31 ± 2.12 mJ/cm^2^ using UV-C LED at 279 nm, as shown in Table 2.

### 3.6. Inactivation of HAV and FCV on Glass Discs Using UV-C at 279 nm

HAV on glass discs showed reduction between 0.5 to 2.2 log PFU after treatment with UV-C LED at 279 nm for 0.17 to 1.0 min (corresponding to doses of 3.28–19.68 mJ/cm^2^), respectively (Table S5 and Figure 3). There was significant reduction in HAV titers after treatment for 0.17 min of 0.5 ± 0.10 log PFU, with 1.3 ± 0.10 and 1.7 ± 0.10 log PFU reduction after 0.5 and 0.67 min, and 2.2 ± 0.20 log PFU reduction after 1 min treatment (*p* ≤ 0.05). The calculated D_10_-value (mJ/cm^2^) for 1 log inactivation of HAV on glass discs was and 8.77 ± 2.08 mJ/cm^2^ using UV-C at 279 nm, as shown in Table 1.

FCV on glass discs showed reduction between 0.93 to 2.57 log PFU after treatment with UV-C LED at 279 nm of 0.17 to 1.0 min (corresponding to doses of 3.28–19.68 mJ/cm^2^), respectively (Table S6 and Figure 4). There was significant reduction in FCV titers after treatments for 0.17 min, 0.33 min, 0.5 min, and 0.67 min of 0.93 ± 0.02, 1.41 ± 0.05, 1.86 ± 0.11, and 2.31 ± 0.19 log PFU, respectively, while tailing occurred after 0.67 min (*p* ≤ 0.05) (Figure 4). The calculated D_10_-value (mJ/cm^2^) for 1-log reduction of FCV on glass discs was 7.88 ± 0.86 mJ/cm^2^ using UV-C LED at 279 nm, as shown in Table 2.

### 3.7. Surface Roughness (Ra) Measurements of Each Model Surface

The average Ra values for stainless-steel coupons, ceramic coupons, and glass discs were calculated to be 7.3, 56.2, and 12.3 μm, respectively. 

## 4. Discussion

Prevention of cross contamination is of utmost concern in the food processing industry as foodborne pathogens can survive on food contact surfaces for extended periods and be transmitted to other items (knives, spoons), food, and surface areas after contact leading to foodborne outbreaks [33]. In addition to surface sanitation with chemical washes, traditional UV-C at 254 nm is commonly used for microbial decontamination of surfaces. Evaluation of the inactivation of *Salmonella* (8–9 log CFU/mL) inoculated on stainless-steel, HDPE, waxed cardboard, and PVC coupons (common surfaces found within the tomato processing industry) using UV-C at 254 nm showed that 2.75, 2.93, 1.39, and 1.91 log CFU reductions, respectively were obtained at doses of 3.3 mJ/cm^2^ [34]. Increased UV-C doses of 19.7 mJ/cm^2^ were shown to result in reductions of 3.51, 4.32, 1.43, and 3.51 log CFU on stainless-steel, HDPE, waxed cardboard, and PVC, respectively, with waxed cardboard showing the least amount of reduction at both tested dosages, as the wax can provide a shielding effect from UV radiation [34,35]. While traditional UV-C systems have been routinely used, surface disinfection using UV-C LED systems including use of wavelengths at 279 nm continue to be investigated. Stainless-steel discs inoculated by droplets containing 7-log CFU/mL of either *Listeria monocytogenes*, *Escherichia coli*, or *Salmonella enterica* serovar Typhimurium after treatments with UV-C LED systems at 279 nm resulted in D_10_-values of 3.02 ± 0.1, 2.70 ± 0.1 mJ/cm^2^, and 1.90 ± 0.1 mJ/cm^2^, respectively (with >3 log CFU/mL inactivation at the highest dosage of 12 mJ/cm^2^) for the tested bacteria [24]. Therefore, this research investigated the ability of UV-C at 254 nm and UV-C LED at 279 nm to inactivate HAV and FCV on model food contact surfaces including stainless-steel, ceramic and glass. 

HAV is known to be resilient and stable to most environmental stressors. Hence, the high D_10_-value (mJ/cm^2^) of 26.05 ± 0.60 mJ/cm^2^ for HAV using UV-C LED at 279 nm and 14.53 ± 2.52 mJ/cm^2^ using UV-C at 254 nm on ceramic surfaces, respectively, is not surprising (*p* < 0.05). This difference in dosage between the two systems could be attributed to the surface roughness and inherent properties of HAV that could result in adherence to ceramic and resistance to UV-C LED at 279 nm. These high parameters were followed by D_10_-values (mJ/cm^2^) of HAV on stainless-steel surfaces of 19.53 ± 2.45 mJ/cm^2^ for UV-C LED at 279 nm and 9.48 ± 0.34 mJ/cm^2^ for UV-C at 254 nm, with HAV on glass discs displaying the lowest D_10_-values (mJ/cm^2^) of 8.77 ± 2.08 mJ/cm^2^ for UV-C LED at 279 nm and 6.91 ± 1.93 mJ/cm^2^ for UV-C at 254 nm, respectively. 

Similarly, FCV on ceramic coupons displayed higher D_10_-values (mJ/cm^2^) of 8.31 ± 2.12 mJ/cm^2^ for UV-C LED at 279 nm and 6.25 ± 1.90 mJ/cm^2^ for UV-C 254 nm, respectively compared to when inoculated on stainless-steel and glass surfaces. Thus, FCV showed a similar trend to HAV with regards to inactivation on ceramic requiring higher doses using UV-C LED at 279 nm than UV-C at 254 nm. However, unlike HAV, FCV on glass discs showed D_10_-values of 7.88 ± 0.86 mJ/cm^2^ with UV-C LED at 279 nm and 4.69 ± 0.03 mJ/cm^2^ with UV-C at 254 nm, and on stainless-steel coupons showed D_10_-values of 7.097 ± 2.11 mJ/cm^2^ for UV-C LED at 279 nm and 3.65 ± 0.06 mJ/cm^2^ for UV-C at 254 nm.

Based on the data obtained, HAV and FCV needed the highest dose for inactivation on contaminated ceramic surfaces by both UV-C systems (~9.84–73.8 mJ/cm^2^ for UV-C LED at 279 nm and ~3.3–48.8 mJ/cm^2^ for UV-C at 254 nm; *p* < 0.05). The high doses of HAV inactivation on ceramic may be associated with surface roughness (as mentioned earlier) and surface properties that may allow for stronger adherence to ceramic, less penetration of UV-C, as well as the resilience of HAV to UV-C inactivation, related to its nucleic acid and capsid protein structure and content. In fact, researchers also state that surface roughness parameters could play a role in decontamination using UV-C systems [24,32]. Therefore, different surfaces may require different doses for microbial inactivation. With ceramic showing a higher surface roughness Ra value of 56.2 µm compared to stainless-steel and glass (7.3 and 12.3 μm, respectively), it is likely that the high Ra values contribute to accumulation of viruses, offering protection against treatments, thus providing higher survival rates and increased dosage requirements, as discussed previously [32]. These researchers compared the inactivation of *E. coli*, *Salmonella enteritidis*, and *Pseudomonas fragi* by UV-C LED at 279 nm on glass, silicone rubber, and stainless-steel surfaces that had surface roughness values 0.020, 0.576, and 1.473 μm with silicone rubber showing the highest resistance to UV-C treatment resulting in only 1.91, 2.91, and 3.08 log CFU/mL reduction for *E. coli*, *Salmonella enteritidis*, and *P. fragi*, respectively. However, the correlation between surface roughness and inactivation abilities is still debatable/unknown, with varying reports among researchers [32].

Other researchers showed that glass displayed the lowest R_a_ and R_q_ values of 0.0204 and 0.0492 compared to values of stainless-steel with R_a_ and R_q_ values of 0.58 and 0.80, which explained the higher bactericidal effect on glass treated with UV-C at 280 nm [36] and is similar to the findings of this study for HAV inactivation. Kim and Kang [36] showed that the level of inactivation of the tested bacterial pathogens varied dependent on surface type, however glass displayed the highest reduction for all pathogens treated with UV-C LED at 280 nm, followed by PVC, stainless-steel, Teflon, and silicon. Kim and Kang [36] showed that treatments with 280 nm UV-C LED at a dose of 2 mJ/cm^2^, caused 0.9–1.44 log reductions of *E. coli*, with highest reduction on glass, while the lowest reduction was obtained on silicon followed by Teflon, stainless-steel and then PVC. These researchers also showed that at a dose of 3 mJ/cm^2^, *S. Typhimurium* and *L. monocytogenes* were reduced by 0.5–1.66 log CFU and 0.5–0.91 log CFU, respectively that followed the same surface resistance trend as *E. coli* [36]. Similarly, the current study found that HAV contaminated on glass displayed the lowest D_10_-value when compared to stainless-steel and ceramic surfaces after treatment with UV-C LED at 279 nm. This could be explained by the ability of the pathogen to adhere to the surfaces as well as a possible back reflection from the stainless-steel coupons that could contribute to the lower D-values of this target microorganism [24].

Currently, literature related to UV-C at 254 nm and 279 nm dose requirements for inactivation of human foodborne viruses including HAV and HuNoV on varied food contact surfaces is limited for comparative purposes to the current study. HAV on Formica coupons was reported to show similar D_10_-values (mJ/cm^2^) using both traditional UV-C at 254 nm (D_10_ = 12.40 ± 1.15 mJ/cm^2^) and UV-C LED at 279 nm (D_10_ =12.39 ± 0.70 mJ/cm^2^) [28]. In the current study, D_10_-values using UV-C LED at 279 nm for the inactivation of HAV was shown to be 19.53 ± 2.45, 26.05 ± 0.60, and 8.77 ± 2.08 mJ/cm^2^ on stainless-steel, ceramic and glass surfaces, respectively. Thus, HAV showed higher resistance to UV-C LED at 279 nm treatments on ceramic, followed by stainless-steel, Formica, and then glass. Using treatments with UV-C at 254 nm, HAV showed D_10_-values of 9.48 ± 0.34, 14.53 ± 2.52, and 6.91 ± 1.93 mJ/cm^2^ on stainless-steel, ceramic and glass respectively, indicating that HAV had higher resistance to UV-C at 254 nm treatments on Formica, followed by stainless-steel that was similar to ceramic, and then glass. Thus, HAV on glass was found to be most sensitive to UV-C LED at 279 nm compared to ceramic, stainless-steel or Formica surfaces. 

Studies with FCV, MNV, echovirus 12 and MS2 on petri dishes showed that doses of 25, 29, 30 and 70 mJ/cm^2^ using traditional UV-C at 254 nm were required to achieve 4-log reduction, respectively, while 85 mJ/cm^2^ was needed for 4 log reduction of intracellular echovirus 12 [37]. While in the current study, using UV-C at 254 nm, HAV required doses of 26.04 mJ/cm^2^ to achieve a 3.50 log PFU/mL reduction on stainless-steel coupons, 29.3 mJ/cm^2^ to achieve a 3.3 log PFU/mL reduction on ceramic coupons, and 13.02 mJ/cm^2^ to achieve a 2.09 log PFU/mL reduction on glass discs (Tables S1–S3). In addition, FCV required doses of 16.28 mJ/cm^2^ to achieve a 4.8 log PFU/mL reduction on stainless-steel surfaces, 9.77 mJ/cm^2^ to achieve a 2.28 log PFU/mL reduction on ceramic surfaces, and 13.02 mJ/cm^2^ to achieve a 2.86 log PFU/mL reduction on glass surfaces, respectively (Tables S4–S6). Based on these results, MS2 requires the highest dose for inactivation on petri dishes compared to HAV and FCV on stainless-steel, ceramic or glass surfaces. These data also indicate that FCV on Petri dishes was more resistant to UV-C at 254 nm requiring higher doses for similar inactivation than FCV on stainless-steel, ceramic or glass.

When we compare the results of a study with TV, a HuNoV surrogate, significant decreases (*p* < 0.05) in RNA copy number (approx. 1 and 2 log reduction in VP1 gene detection) after 220-nm irradiation treatments at doses between 22.5 and 37.5 mJ/cm^2^, or with 254 nm irradiation at doses of 30 or 37.5 mJ/cm^2^, respectively were reported [10]. Previous research showed that FCV on Formica surfaces was reduced by 2.26 log PFU/mL at a dose of 21.81 mJ/cm^2^, while TV showed reduction of 1.50 log PFU/mL at a dose of 10.91 mJ/cm^2^ [28], which were lower than those reported for RNA copy reduction. In the current study, FCV on stainless-steel, ceramic, and glass coupons treated with UV-C at 254 nm showed reduction of 4.8, 2.67, and 2.86 log PFU/mL at dosages of 16.28, 9.77, and 13.02 mJ/cm^2^, respectively, while HAV on stainless-steel, ceramic, and glass was reduced by 3.63, 3.35, and 2.09 log PFU/mL at dosages of 32.55, 48.83, and 13.02 mJ/cm^2^, respectively. Thus, HAV required higher dosages for inactivation on all tested surfaces compared to FCV on Formica, ceramic, and stainless-steel. The results of this study show that on the tested surfaces, HAV was more resistant to both UV-C systems compared to FCV.

When we compare the inactivation of the pandemic causing SARS-CoV-2 (an enveloped virus at 10^5^ PFU/mL) on contaminated synthetic leather and clothing fabric (porous) and glass, stainless-steel, ceramics, and oaks (non-porous) surfaces by UV-C at 254 nm, doses of 132 mJ/cm^2^ was needed for 1-log reduction, 264 mJ/cm^2^ for a 2-log reduction, and 396 mJ/cm^2^ for a 3 log reduction on porous surfaces, while for all non-porous samples, >3 log reduction was achievable at a dose of 8 mJ/cm^2^, with exposure times all below 1 min (ordered by oak, glass, ceramics, and stainless-steel for shortest to longest exposure time) [38]. The current study showed that for HAV and FCV on all tested non-porous surfaces (stainless-steel, ceramic, and glass) doses ranging from ~7–9 mJ/cm^2^ for HAV and ~3–6 mJ/cm^2^ for FCV were required to achieve a 1-log reduction using UV-C at 254 nm. In addition, this current study showed that the tested virus required higher doses using UV-C LED at 279 nm ranging from 7.097 to 26.05 mJ/cm^2^ than UV-C at 254 nm to achieve a 1-log reduction. Since, HAV and FCV are both non-enveloped viruses, theoretically higher doses for their inactivation would be needed.

When using traditional UV-C at 254 nm, tailing occurred for the inactivation of HAV on stainless-steel and ceramic coupons with doses > 19.53 and 29.30 mJ/cm^2^ (Figure 1), respectively and doses > 6.51 and 8.68 mJ/cm^2^ for FCV on ceramic and glass surfaces (Figure 2), respectively proving ineffective at further viral inactivation. In comparison, for treatments with UV-C LED at 279 nm, tailing was not observed, except for the inactivation of FCV on glass discs with doses > 13.12 mJ/cm^2^ that were ineffective at further viral inactivation (Figure 4). When observing tailing effects in cases such as UV-C inactivation of HAV on stainless-steel and ceramic surfaces at 254 nm or FCV inactivation on ceramic or glass surfaces by UV-C at 254 nm and also glass with UV-C LED at 279 nm, it is important to recognize that the linear model may not be most suitable for prediction of >2 or 3 log reduction. 

As HAV showed the highest D-values using UV-C LED at 279 nm on ceramic coupons (which was significantly higher than on glass surfaces), the differences between the dosage requirements for inactivation of HAV on glass could be associated with the inherent properties of the virus that allow for attachment to the surface (as stated earlier) as well as the properties (structure and components) of the capsid and nucleic acid and the limitation of lower penetration power by UV-C light. 

When comparing reductions of FCV and TV on Formica by UV-C at 279 nm with HAV and FCV on stainless-steel, ceramic, and glass surfaces, FCV and TV on Formica showed reductions of 2.45 and 1.83 log PFU/mL at dosages of 54.6 and 27.3 mJ/cm^2^ [28]. This study showed that FCV on stainless-steel, ceramic, and glass surfaces using UV-C LED at 279 nm was reduced by 3.89, 3.12, and 2.57 log PFU at dosages of 24.6, 24.6, and 19.68 mJ/cm^2^, respectively, while HAV showed reduction of 2.75, 3.37, and 2.2 log PFU/mL at dosages of 49.2, 73.8, and 19.68 mJ/cm^2^, respectively. Inactivation of HAV on ceramic coupons by UV-C LED at 279 nm required a higher dose to achieve >3 log reduction, similar to the inactivation of TV on Formica which required a dose of 54.6 mJ/cm^2^ to achieve a >1.8 log reduction, compared to inactivation of FCV on all surfaces [28]. As reported above, the resistance could be associated with TV having different binding sites for the surfaces based on its capsid structure making it more resistant to UV-C and preventing damage to the capsid proteins (UV-C at 279 nm) or RNA (UV-C at 254 nm). Also, protein-RNA cross-linking or energy transfer from proteins to RNA is reported to occur at UV-C at 210–240 nm and may also occur at UV-C at 279 nm [28].

Recently, FCV on stainless-steel surfaces was shown to be reduced by 3.3 log PFU/disc at a dose of 27.5 mJ/cm^2^ using UV-C LED at 269 nm [27]. In comparison, the current study reported that FCV treated by UV-C LED at 279 nm on stainless-steel, ceramic, and glass surfaces could be decreased 3.89, 3.12, and 2.57 log PFU with doses of 24.6, 49.2, and 19.68 mJ/cm^2^, respectively (Tables S4–S6). Thus, the results of the current study are comparable to that reported by Mariita et al. [27] for stainless-steel surfaces given the differences in the wavelengths used. These UV-C LED systems that potentially target the viral capsid structure can cause damage to proteins due to absorption of UV-C by amino acids around 279 nm, with further oxidation and eventual damage to the nucleic acid, while typically 254 nm UV-C is known to cause dimerization of nucleic acids, nucleic acid damage and thus prevention of viral replication [16,24]. 

Furthermore, UV-C LED systems with 266, 270, 275, and 279 nm were shown to cause 5 to 6 log reduction of *E. coli* O157:H7, *Salmonella enterica* serovar Typhimurium, and *L. monocytogenes* (~10^8^–10^9^ CFU/mL) with low doses of 0.7 mJ/cm^2^ in media within Petri dishes using 279 nm UV-C LED, while inoculated sliced cheese required higher doses of 1, 2, and 3 mJ/cm^2^ for inactivation [39]. *Salmonella* cocktails consisting of *Salmonella* Typhimurium, *S*. Newport, *S*. Enteritidis, *S*. Senftenberg, and *S*. Heidelberg (6.5 log CFU/mL) were shown to be reduced by 1.97 and 3.48 log CFU on stainless-steel surfaces using UV-C LED at 260 to 280 nm at a dose of 2 and 4 mJ/cm^2^, while reductions of 4.74 and 5.2 log CFU were observed on HD polyethylene for irradiances of 2 and 4 mJ/cm^2^ [40]. Though, the *Salmonella* cocktail on stainless-steel surfaces was rapidly reduced by 1.3 log CFU/mL after the initial dose of 30 mJ/cm^2^, a maximum reduction of 1.97 log CFU/mL after the final dose of 120 mJ/cm^2^ was obtained, similar to the tailing observed in the current study with viruses [40]. Thus, the bacterial pathogens required lower doses for inactivation compared to the higher doses required for inactivation of the HAV and FCV used in the current study on food contact surfaces. 

Overall, this study provided the target doses needed for 1-log inactivation of HAV and FCV when dried on three food contact surfaces using UV-C at 254 nm and UV-C LED at 279 nm based on the linear model. This data lays the foundation for the food industry to design systems needed to deliver the desired doses to inactivate the target pathogens on food contact surfaces.

## 5. Conclusions

This research shows the promising application of UV-C LED at 279 nm for the inactivation of foodborne viruses, HAV and the tested HuNoV surrogate, FCV, on dried model food contact surfaces (stainless-steel, ceramic, and glass). This study helped bridge the gaps in knowledge related to the dose requirements for inactivation of HAV and FCV using both traditional UV-C at 254 nm and UV-C LED at 279 nm. The results also showed notable increased resistance of HAV compared to FCV to UV-C inactivation on the tested surfaces based on the linear model D_10_-values. While successful at achieving similar levels of inactivation, UV-C LED at 279 nm requires higher doses for inactivation of both HAV and FCV regardless of surface type. This UV-C dose data will be useful in laying the foundation for the design of UV-C LED systems to deliver the optimal doses needed to inactivate foodborne viruses on surfaces in order to decrease the risk of viral contamination. However, it is important to note that only linear models were used in this study that may not be the best fit if and when tailing effects are observed. Future studies are focused on determination of the UV-C dose requirements of aerosolized viral deposits and aerosolized viruses, as well as understanding the effect of temperature, relative humidity, organic load, and varied wavelengths of UV-C LED systems for viral inactivation on surfaces for practical applications to protect public health. 

## Figures and Tables

**Table 1:** D_10_-values (mJ/cm^2^) and D-values (min) of HAV treated with either UV-C (254 nm) or UV-C LED (279 nm) on model food contact surface coupons.

Surface Type	UV-C LED System (279 nm) D_10_-Value (mJ/cm^2^)	UV-C LED System (279 nm) D-Value (min)	UV-C (254 nm) D_10_-Value (mJ/cm^2^)	UV-C (254 nm) D-Value (min)
Stainless-steel	19.53 ± 2.45 ^Ab^	1.0 ± 0.12 ^Ab^	9.48 ± 0.34 ^Bb^	0.73 ± 0.03 ^Bb^
Ceramic	26.04 ± 0.60 ^Aa^	1.3 ± 0.03 ^Aa^	14.53 ± 2.52 ^Ba^	1.1 ± 0.12 ^Ba^
Glass	8.77 ± 2.08 ^Ac^	0.47 ± 0.11 ^Bc^	6.91 ± 1.93 ^Bb^	0.56 ± 0.16 ^Ab^

Capital letters denote statistically significant differences when compared across one row between UV-C systems (*p* < 0.05). Lowercase letters denote statistically significant differences when compared down a treatment medium (one column) (*p* < 0.05). Data are reported as averages of triplicate treatments ± standard deviations; Note: both optical devices have different UV intensities.

**Table 2:** D_10_-values (mJ/cm^2^) and D-values (min) of FCV treated with either UV-C (254 nm) or UV-C LED (279 nm) on three model food contact surface coupons.

Surface Type	UV-C LED System (279 nm) D_10_-Value (mJ/cm^2^)	UV-C LED System (279 nm) D-Value (min)	UV-C (254 nm) D_10_-Value (mJ/cm^2^)	UV-C (254 nm) D-Value (min)
Stainless-steel	7.097 ± 2.11 ^Aa^	0.38 ± 0.11 ^Aa^	3.65 ± 0.06 ^Ba^	0.28 ± 0.001 ^Ba^
Ceramic	8.31 ± 2.12 ^Aa^	1.37 ± 0.34 ^Ba^	5.92 ± 1.90 ^Ba^	1.45 ± 0.48 ^Aa^
Glass	7.82 ± 0.86 ^Aa^	0.40 ± 0.05 ^Aa^	4.69 ± 0.03 ^Ba^	0.36 ± 0.001 ^Ba^

Capital letters denote statistically significant differences when compared across one row between UV-C systems (*p* < 0.05). Lowercase letters denote statistically significant differences when compared down a treatment medium (one column) (*p* < 0.05). Data represent averages of triplicate treatments ± standard deviations; both optical devices have different UV intensities.

## Data Availability

The data obtained for this study are included in the article, and any further information can be obtained from the corresponding author.

## Supplementary Materials

The following supporting information can be downloaded at: https://www.mdpi.com/article/10.3390/foods13182892/s1, Table S1: Inactivation of HAV treated with either UV-C (254 nm) or UV-C LED (279 nm) on Stainless-steel coupons; Table S2: Inactivation of FCV treated with either UV-C (254 nm) or UV-C LED (279 nm) on Stainless-steel coupons; Table S3: Inactivation of HAV treated with either UV-C (254 nm) or UV-C LED (279 nm) on Ceramic coupons; Table S4: Inactivation of FCV treated with either UV-C (254 nm) or UV-C LED (279 nm) on Ceramic coupons; Table S5: Inactivation of HAV treated with either UV-C (254 nm) or UV-C LED (279 nm) on glass discs; Table S6: Inactivation of FCV treated with either UV-C (254 nm) or UV-C LED (279 nm) on glass discs.
