# Supplementary material for: Inactivation of Hepatitis A Virus and Feline Calicivirus on Model Food Contact Surfaces by Ultraviolet Light (UV-C) Systems
Source: Foods. 2024 Sep 12;13(18):2892. doi: 10.3390/foods13182892 (PMC11430824; doi:10.3390/foods13182892)
Supplement: Supplementary file 1 [file foods-13-02892-s001.zip › foods-3194871-supplementary.pdf]

Supplementary Table S1. Inactivation of HAV treated with either UV-C (254 nm) or UV-C LED (279 nm) on Stainless-steel coupons.

Time (mins)	UV-C LED system (279 nm) UV dose (mJ/cm^2)	UV-C LED system (279 nm) Reduction (log PFU/mL)	UV-C (254 nm) UV dose (mJ/cm2)	UV-C (254 nm) Reduction (log PFU/mL)
0	0	0^{A}	0	0^{A}
0.5	9.84	1.20 ± 0.07^{B}	6.51	1.26 ± 0.07^{B}
1.0	19.68	1.90 ± 0.12^{BC}	13.02	2.53 ± 0.05^{C}
1.5	29.52	1.96 ± 0.02^{C}	19.53	2.99 ± 0.29^{CD}
2.0	39.36	2.31 ± 0.15^{CD}	26.04	3.50 ± 0.16^{D}
2.5	49.2	2.75 ± 0.15^{D}	32.55	3.63 ± 0.09^{D}

*Capital letters denote statistically significant differences when compared across treatment times ($p < 0.05$). Data are reported as averages of triplicate treatments ± standard deviations.

Supplementary Table S2 Inactivation of FCV treated with either UV-C (254 nm) or UV-C LED (279 nm) on Stainless-steel coupons.

Time (mins)	UV-C LED system (279 nm) UV dose (mJ/cm^2)	UV-C LED system (279 nm) Reduction (log PFU/mL)	UV-C (254 nm) UV dose (mJ/cm2)	UV-C (254 nm) Reduction (log PFU/mL)
0	0	0^{A}	0	0^{A}
0.25	4.92	1.93 ± 0.05^{B}	3.26	1.1 ± 0.04^{B}
0.5	9.84	1.94 ± 0.04^{B}	6.51	2.0 ± 0.10^{C}
0.75	14.76	2.99 ± 0.11^{C}	9.77	2.5 ± 0.20^{D}
1.0	19.68	3.17 ± 0.22^{C}	13.02	3.3 ± 0.10^{E}
1.25	24.6	3.89 ± 0.19^{D}	16.28	4.8 ± 0.0^{F}

*Capital letters denote statistically significant differences when compared across treatment times ($p < 0.05$). Data denote Averages of triplicate treatments ± standard deviations

Supplementary Table S3. Inactivation of HAV treated with either UV-C (254 nm) or UV-C LED (279 nm) on Ceramic coupons.

Time (mins)	UV-C LED system (279 nm) UV dose (mJ/cm^2)	UV-C LED system (279 nm) Reduction (log PFU/mL)	UV-C (254 nm) UV dose (mJ/cm2)	UV-C (254 nm) Reduction (log PFU/mL)
0	0	0^A	0	0^A
0.75	14.76	1.63 ± 0.17^B	9.77	1.52 ± 0.12^B
1.5	29.52	1.95 ± 0.12^B	19.53	2.32 ± 0.11^C
2.25	44.28	2.52 ± 0.11^C	29.3	3.30 ± 0.13^D
3.0	59.04	2.47 ± 0.12^C	39.06	3.43 ± 0.12^D
3.75	73.8	3.37 ± 0.10^D	48.83	3.35 ± 0.09^D

*Capital letters denote statistically significant differences when compared across treatment times ($p < 0.05$). Data denote Averages of triplicate treatments ± standard deviations.

Supplementary Table S4. Inactivation of FCV treated with either UV-C (254 nm) or UV-C LED (279 nm) on Ceramic coupons.

Time (mins)	UV-C LED system (279 nm) UV dose (mJ/cm^2)	UV-C LED system (279 nm) Reduction (log PFU/mL)	UV-C (254 nm) UV dose (mJ/cm2)	UV-C (254 nm) Reduction (log PFU/mL)
0	0	0^A	0	0^A
0.25 (UV-C 254)			3.26	0.77 ± 0.07^B
0.5	9.84	$1.10 \pm 0.04^{B.}$	6.51	$1.46 \pm 0.0.03^C$
0.75 (UV-C 254)			9.77	2.28 ± 0.07^D
1.0	19.68	2.27 ± 0.06^B	13.02	2.40 ± 0.09^D
1.25 (UV-C 254)			16.28	2.67 ± 0.12^D
1.5 (UV-C 279)	29.52	2.51 ± 0.07^C		
2.0 (UV-C 279)	39.36	2.73 ± 0.11^C		
2.5 (UV-C 279)	49.2	3.12 ± 0.11^D		

*Capital letters denote statistically significant differences when compared across treatment times ($p < 0.05$). Data denote Averages of triplicate treatments ± standard deviations

Supplementary Table S5. Inactivation of HAV treated with either UV-C (254 nm) or UV-C LED (279 nm) on glass discs.

Time (mins)	UV-C LED system (279 nm) UV dose (mJ/cm^2)	UV-C LED system (279 nm) Reduction (log PFU/mL)	UV-C (254 nm) UV dose (mJ/cm2)	UV-C (254 nm) Reduction (log PFU/mL)
0	0	0^A	0	0^A
0.17	3.28	0.5 ± 0.10^B	2.17	0.85 ± 0.02^B
0.33	6.56	0.9 ± 0.10^B	4.34	$1.3 \pm 0.10B^C$
0.5	9.84	1.3 ± 0.10^C	6.51	1.5 ± 0.04^{CD}
0.67	13.12	1.7 ± 0.10^D	8.68	1.6 ± 0.07^{CD}
1.0	19.68	2.2 ± 0.20^E	13.02	2.09 ± 0.10^D

*Capital letters denote statistically significant differences when compared across treatment times ($p < 0.05$). Data denote Averages of triplicate treatments ± standard deviations.

Supplementary Table S6. Inactivation of FCV treated with either UV-C (254 nm) or UV-C LED (279 nm) on glass discs.

Time (mins)	UV-C LED system (279 nm) UV dose (mJ/cm^2)	UV-C LED system (279 nm) Reduction (log PFU/mL)	UV-C (254 nm) UV dose (mJ/cm^2)	UV-C (254 nm) Reduction (log PFU/mL)
0	0	0^{A}	0	0^{A}
0.17	3.28	0.93 ± 0.02^{B}	2.17	0.98 ± 0.07^{B}
0.33	6.56	1.41 ± 0.05^{C}	4.34	1.47 ± 0.09^{C}
0.5	9.84	1.86 ± 0.11^{D}	6.51	2.04 ± 0.03^{D}
0.67	13.12	2.31 ± 0.19^{E}	8.68	2.45 ± 0.06^{E}
1.0	19.68	2.57 ± 0.07^{E}	13.02	2.86 ± 0.07^{E}

*Capital letters denote statistically significant differences when compared across treatment times ($p < 0.05$).
Data denote Averages of triplicate treatments ± standard deviations
